# The physical activity of children and adolescents in Germany 2003-2017: The MoMo-study

**DOI:** 10.1371/journal.pone.0236117

**Published:** 2020-07-16

**Authors:** Steffen C. E. Schmidt, Bastian Anedda, Alexander Burchartz, Doris Oriwol, Simon Kolb, Hagen Wäsche, Claudia Niessner, Alexander Woll

**Affiliations:** Karlsruhe Institute of Technology, Karlsruhe, Germany; La Inmaculada Teacher Training Centre (University of Granada), SPAIN

## Abstract

With digitalization and virtual entertainment being the megatrends of the 21st century, there is reasonable concern about the role of physical activity (PA) in the daily life of children and adolescents. To identify risk-groups with insufficient PA and to guide interventions, continuous and representative tracking of PA is crucial. In this paper, representative PA data of children and adolescents from the Motorik-Modul (MoMo) baseline study (2003–2006, N = 4,528) is compared to those of Wave 2 (2014–2017, N = 3,708). Participants aged 4–17 were drawn out of 167 sample points in Germany and the data was weighted to ensure representativeness for Germany. Organized (sports clubs and schools) and unorganized (unorganized sports and playing outside) PA was measured by questionnaire and stratified by sex, age, and socioeconomic status. Contrary to common expectation, overall PA remained stable among youths in the past ten years, however, there is an ongoing trend towards organized forms of PA at the expense of unorganized sports and playing outside. Besides different trends in settings, there is inequality in PA distribution among socioeconomic status and gender, unequally pronounced in different settings.

## 1 Introduction

A multitude of health benefits caused by physical activity (PA) is well documented [1]. It is also known that an active lifestyle during childhood reduces the risk for several diseases in adulthood [2, 3] and the World Health Organization (WHO) promotes PA as part of a healthy lifestyle, particularly in children [4]. Although reviews and meta-analyses still proclaim a global "childhood physical activity crisis" [5, 6] recent studies point to a small increase in engagement in sports and PA of children and adolescents since the early 2000s in Germany [7, 8] paralleled by a global increase in screen-time [9]. The official numbers regarding sports clubs from the German Olympic Sports Association (DOSB) support these findings [10]. To explain this development, different reasons have been stated such as more variety in sports clubs and a broader range of extracurricular sports activities in schools [8]. This trend may appear positive at first glance, however, overall PA in Germany is still on an insufficient level. Experts in Germany graded the overall PA among children and adolescents living in Germany with "D-", stating that about only 20% of boys and girls in Germany reach 60 minutes of moderate-to-vigorous PA per day [11]. With increasing, yet insufficient amounts of PA among youth, the identification and promotion of high-risk-groups for physical inactivity becomes more important.

Besides some variables that are consistently associated with children’s PA such as perceived barriers, perceived activity competence, program/facility access and parental or peer-group support [12], education and the socioeconomic status (SES) are the most dominant predictors of PA among peers in Germany [13, 14]. In a large meta-analysis, Althoff et al. analyzed 68 million days of PA from 717,527 people across 111 countries all over the world and found, that inequalities in how PA is distributed among countries are a better predictor for obesity than the amount of PA itself [15]. For example, the authors found that the United States and Mexico have similar average daily steps (4774 vs. 4692) but the inequality among its distribution measured by the Gini-Coefficient [16] is much higher in the US. The authors state that this may be an explanation for the fact that the prevalence of obesity is 27,7% in the US and "only" 18.1% in Mexico.

Knowing about social and environmental circumstances that put children and adolescents at risk for physical inactivity and enhance inequality throughout a country is crucial in designing tailored interventions. Therefore, it is important to track and report detailed, comparable information about PA, periodically, and from representative samples. In Germany, the PA of children and adolescents is tracked nationwide and representative by the Motorik-Modul Study (MoMo) [17] and merged with health-related data from the KiGGS study [18] by the Robert Koch Institute (RKI). In this paper, we compare PA measured by questionnaire in different settings between two representative cross-sectional samples of children and adolescents, 2003–2006 (baseline) and 2014–2017 (Wave 2), and discuss inequalities in distribution among SES, age, and gender.

## 2 Materials and methods

### 2.1 Sample and representativeness

MoMo is a nationwide study on physical fitness, PA and health in children and adolescents, and part of the German Health Interview and Examination Survey for Children and Adolescents, KiGGS [17, 18]. To maximize representativeness, a nationwide, stratified, multi-stage sample was drawn during each Wave [19]. First, a systematic sample of 167 primary sampling units was selected from an inventory of German communities stratified according to the BIK classification system that measures the level of urbanization and geographic distribution [20]. The probability of any community being picked was proportional to the number of inhabitants younger than 18 years in that community. Second, an age-stratified sample was drawn from the official registers of residents. The final number of participants aged 4–17 years was N = 4,528 at Baseline (35.8% response, age 4–5: N = 989; age 6–10: N = 1712; age 11–13: N = 886; age 14–17: N = 941) and 3,708 in Wave 2 (33.2% response, age 4–5: N = 321; age 6–10: N = 1343; age 11–13: N = 954; age 14–17: N = 1088).

A weighting procedure was used to account for potential bias in outcome variables caused by selective unit nonresponse [19, 21]. In a first step, inverse probability weights were applied via logistic regression to eliminate differences in outcome variables between the MoMo sample and the larger KiGGS cohort. In the second step, the MoMo subsample was stratified using data of the German Micro Census 2004 and 2014 to ensure the representativeness of the target population (children and adolescents aged 4–17 years living in Germany) regarding sex, age, region, migration background, and education level.

MoMo baseline data was collected between 2003 and 2006 and MoMo Wave 2 data between 2014 and 2017. Parents and adolescents were invited to examination rooms at central locations within proximity to their homes in the 167 cities and municipalities in which the study was conducted.

This study was approved by the ethics committee of the State Chamber of Physicians of Baden-Wuerttemberg (Germany). The KiGGS and the MoMo studies were approved by the Charité/Universitätsmedizin Berlin ethics committee and the Federal Office for the Protection of Data and were conducted according to the Declaration of Helsinki. All participants of the MoMo study gave their written consent to participate and were informed in detail about the study and data management by the Robert Koch Institute. Parents gave their written consent for minors and the presence of a legal guardian was mandatory under the age of 15.

### 2.2 PA assessment

The MoMo PA Questionnaire (MoMo-PAQ) was used to assess self-reported habitual PA in adolescents in different settings (sports clubs, leisure time, and school) [22]. The MoMo-PAQ consists of 28 items and measures frequency, duration, intensity, and setting of PA in a normal week, without a defined reference period. Data obtained with the MoMo-PAQ are sufficiently reliable (test-retest reliability: ICC  =  0.68) [22].

PA in school was assessed by two items about the frequency (times per week) of 45-minute classes in curricular and extracurricular sports activities. Calculated total minutes were multiplied with a correction factor of 8.5 divided by 12 to compensate for vacations.

Club sport was assessed by four items: type of club sport activity, duration (minutes per session), frequency (times per week) of each activity, and time throughout the year (months per year) of each activity. From those items, club sport minutes per week were calculated.

Unorganized, leisure-time sports activity was assessed by three items: type, duration (minutes per week), and time throughout the year (months per year). These items were combined in an index reflecting minutes per week at leisure-time unorganized sports activities.

Unorganized playing outside was assessed by an 8-scaled item about days per week in which the child plays outside (“How often do you normally play outside during a week (for example: playing tag, skipping rope or going to the swimming pool)”: 0–7 days per week).

Types of sports that do not lead to an increase in energy expenditure using large skeletal muscles (for example esports) were not defined as PA according to the definition of Caspersen, Powell & Christenson [23], and minutes were multiplied with zero.

### 2.3 Socioeconomic status

Individual-level SES included items on educational and professional status and total household income and was calculated separately for both parents with the higher score being used [24]. Adolescents with separated parents were assigned the socioeconomic status of the parent they lived with. The three aspects income, educational and professional status were scored on a scale from 1 to 7 and a sum-score was created (range: 3–21) and categorized into low (3–8), medium (9–14) and high (15–21) socioeconomic status [25].

### 2.4 Statistics

All statistical tests were conducted using IBM SPSS 25 (IBM Corporation, Armonk, NY). Statistically significant differences between Baseline and Wave 2 were determined via 95% confidence intervals (95% CI) for complex samples. The SPSS complex sample procedure accounts for weighting and the fact that MoMo is a cluster sample of 167 sample points in Germany. Statistical significance (p < .05) was assumed when both 95% CIs did not include the mean of their counterpart. Means and standard deviations are reported for PA in different settings. Means and 95% CIs are reported for overall PA. Data is presented weighted to ensure representativeness. Univariate ANOVAs were calculated to reveal significant differences and interactions in the amount of PA between sex and SES.

## 3 Results

The most important settings for PA among children and adolescents in Germany are schools, sports clubs, and unorganized sports during leisure time. Table 1 shows the attendance in PA regarding curricular sports, extracurricular sports, sports clubs, and unorganized sports at Baseline and Wave 2 stratified for sex and age.

**Table 1:** Attendance in PA of children and adolescents in Germany.

		Curricular sports		Extracurricular sports		Sport club		Unorganized sports		Playing outside	
										> 3 days/week	
		Baseline	Wave 2	Baseline	Wave 2	Baseline	Wave 2	Baseline	Wave 2	Baseline	Wave 2
		N = 4,508	N = 3,667	N = 4,520	N = 3,667	N = 4,492	N = 3,624	N = 4,527	N = 3,599	N = 4,524	N = 3,590
4–5	m	77.4%	90.7%	-	-	43.3%	51.2%	42.0%	23.1*%	91.3%	93.3%
	f	78.6%	89.9%	-	-	50.3%	49.5%	38.9%	26.3*%	83.4%	89.0%
	Ø	78.0%	90.3%	-	-	46.8%	50.4%	40.5%	24.7*%	87.4%	91.2%
6–10	m	94.3%	97.0%	8.6%	37.7*%	65.6%	73.3*%	53.9%	26.6*%	82.1%	76.8%
	f	93.6%	97.6%	11.0%	33.9*%	51.4%	66.2*%	54.4%	37.3*%	77.3%	83.0%
	Ø	93.9%	97.3%	9.8%	35.9*%	58.7%	69.9*%	54.1%	31.8*%	79.8%	79.8%
11–13	m	97.9%	99.7*%	14.1%	32.9*%	64.3%	65.3%	60.9%	33.5*%	64.4%	53.5*%
	f	98.3%	99.5*%	14.0%	23.4*%	49.1%	58.4%	61.4%	36.2*%	59.5%	46.5*%
	Ø	98.1%	99.6*%	14.1%	28.3*%	56.9%	61.9%	61.1%	34.8*%	62.0%	50.1*%
14–17	m	89.1%	86.8%	10.8%	12.8%	54.0%	56.8%	64.5%	49.3*%	41.9%	18.2*%
	f	90.6%	88.4%	11.2%	9.5%	43.3%	46.4%	55.6%	46.1*%	32.2%	10.8*%
	Ø	89.8%	87.6%	11.0%	11.2%	48.8%	51.8%	60.1%	47.7*%	37.2%	14.6*%
4–17	m	91.2%	95.2%	10.8%	23.9*%	58.7%	63.6*%	57.3%	34.5*%	66.5%	57.2*%
	f	91.7%	95.8%	11.8%	19.5*%	48.1%	56.2*%	54.2%	38.2*%	59.7%	54.9%
	Ø	91.4%	93.8%	11.3%	21.8*%	53.5%	60.0*%	55.8%	36.3*%	63.1%	56.1*%

m: male; f: female, Ø: mean of males and females

*: Significant difference (p < .05) between Baseline (2003–2006) and Wave 2 (2014–2017)

The engagement in extracurricular sports and sports clubs significantly increased from Baseline to Wave 2, whereas the engagement in unorganized sports significantly decreased. Whereas unorganized sports decreased among all ages and for both, boys and girls, organized PA mainly increased for 6–13-year olds regarding extracurricular sports and 6–10-year olds regarding sports clubs.

Table 2 shows that the overall amount of PA among 4–17-year-olds remained relatively stable from 2003 to 2017. Only girls aged 4–5 and 6–10 show significant positive changes. However, PA's allocation among settings did change over time. The amount of PA in extracurricular sports significantly increased from 4.6 to 23.9 minutes per week among 6-10-year-olds, from 8.3 to 26.0 minutes per week among 11-13-year-olds and from 6.3 to 9.3 minutes per week among 14-17-year-olds with no relevant differences among genders. Regarding PA in sports clubs, the overall increase was higher among girls (13.4 minutes per week) than in boys (6.6 minutes per week). Considering unorganized sports activities, PA decreased significantly from 83.1 to 52.5 minutes per week with significant changes among 4-13-year-olds of both sexes.

**Table 2:** Amount of PA in minutes of children and adolescents in Germany.

age	sex	Curricular sports (mean±s)		Extracurricular sports (mean±s)		Sport club (mean±s)		Unorganized sports (mean±s)		Total amount of sports (mean (95% CI))	
		Baseline	Wave 2	Baseline	Wave 2	Baseline	Wave 2	Baseline	Wave 2	Baseline	Wave 2
		N = 4,508	N = 3,667	N = 4,520	N = 3,667	N = 4,492	N = 3,624	N = 4,527	N = 3,599	N = 4,491	N = 3,557
4–5	m	45.1±35.1	49.5±38.1	0.0±0.0	0.0±0.0	36.3±54.2	42.1±52.4*	54.3±102.0	28.4±107.4*	129.5 (115.6–143.4)	122.0 (94.7–149.2)
	f	49.3±41.3	54.9±40.4	0.0±0.0	0.0±0.0	41.3±51.3	34.5±40.9*	42.8±103.0	18.9±37.3*	125.5 (111.8–139.2)	107.8 (90.7–124.9)*
	Ø	47.1±38.3	52.2±39.3	0.0±0.0	0.0±0.0	38.8±52.8	38.3±47.1	48.7±102.6	23.8±81.3*	127.5 (117.0–138.0)	114.9 (99.1–130.7)
6–10	m	79.5±27.4	79.1±32.3	5.1±18.3	24.2±37.1*	93.2±96.4	106.3±97.6*	76.6±123.7	34.8±89.3*	251.1 (236.9–265.3)	243.1 (223.9–262.2)
	f	76.2±27.0	80.3±30.5	4.0±17.4	23.6±39.1*	57.8±76.4	86.4±89.3*	70.0±111.1	35.4±94.3*	203.9 (189.6–218.3)	226.5 (208.9–244.1)*
	Ø	77.9±27.2	79.7±31.4	4.6±17.8	23.9±38.1*	76.0±89.0	96.7±94.2*	73.3±117.7	35.1±91.7*	228.1 (217.6–238.7)	235.0 (221.9–248.1)
11–13	m	81.9±26.6	89.5±38.0	8.2±24.9	29.3±54.8*	133.0±144.0	135.7±137.6	114.1±169.6	54.7±120.0*	335.1 (309.2–361.0)	312.3 (282.2–342.5)
	f	78.9±24.3	84.2±28.1	8.4±24.7	22.6±54.0*	79.7±112.1	100.6±126.4*	83.2±136.4	59.7±162.0*	249.6 (225.0–274.1)	266.2 (227.8–304.5)
	Ø	80.5±25.6	87.0±33.7	8.3±24.8	26.0±54.5*	107.2±132.2	118.6±133.3*	99.0±155.0	57.1±141.9*	293.6 (275.5–311.6)	289.8 (263.8–315.7)
14–17	m	67.5±28.9	65.2±34.8	6.7±22.2	10.5±34.9*	138.3±177.5	143.6±177.7	115.5±179.3	90.3±142.2	325.2 (297.5–352.8)	312.5 (270.4–354.5)
	f	65.0±26.1	64.1±33.0	6.0±21.7	8.0±28.3	83.4±131.3	85.8±131.3	76.7±129.2	73.3±149.9	229.2 (210.3–248.1)	230.8 (198.9–262.6)
	Ø	66.3±27.6	64.7±33.9	6.3±21.9	9.3±31.9*	111.5±159.0	115.4±159.4	96.6±158.0	82.0±146.1	278.4 (260.9–295.8)	272.9 (247.0–298.7)
4–17	m	72.2 ±30.9	73.1±37.4	6.4±21.5	17.8±39.9*	109.2±138.9	115.4±135.7	94.3±153.3	55.1±119.1*	277.9 (265.3–290.3)	262.6 (245.6–279.6)
	f	70.0±29.7	72.6±34.1	5.8±21.0	15.4±38.4*	68.6±103.8	82.0±109.6*	71.4±122.4	49.8±126.7*	211.7 (201.5–221.9)	219.6 (204.1–235.2)
	Ø	71.1±30.3	72.9±35.8	6.1±21.3	16.6±39.2*	89.4±124.7	99.1±124.8*	83.1±139.6	52.5±122.8*	245.5 (236.5–254.6)	241.7 (230.2–253.1)

m: male; f: female, Ø: mean of males and females; s: standard deviation; 95% CI: 95% confidence intervals for complex samples

*: Significant difference (p < .05) between Baseline and Wave 2

Overall PA increases with age until it peaks at the age of 11–13 and decreases again at the threshold to adulthood. Differences between boys and girls are especially prevalent in sports clubs with significant differences between boys and girls aged 6–10, 11–13, and 14–17 at Baseline and Wave 2 and have slightly reduced regarding unorganized sports from Baseline to Wave 2 (Table 2). The total amount of sports significantly differs between boys and girls aged 6–10 at Baseline and between boys and girls aged 11–13 and 14–17 at both measurement points.

Table 3 shows the amount of PA in different settings stratified by sex and SES in Wave 2. Gender differences are significant for extracurricular sports (p = .01), sports clubs (p < .01), and unorganized sports (p < .01), but not for curricular sports (p = .49). The overall amount of sports increases significantly with higher SES, which is particularly prevalent in the setting sports club. Unorganized sports (p = .60) and curricular sports (p = .21) do not differ significantly with regard to SES. PA in extracurricular sports is higher among those with a low SES. Significant interactions between sex and SES for the total amount of sports (p < .01) and unorganized sports (p < .01) finally reveal that girls from families with a low SES show the least amount of PA from sports and in contrast to their male counterparts, do engage less in unorganized sports.

**Table 3:** Amount of different types of PA in minutes stratified by socioeconomic status (SES) and sex.

		Curricular sports (mean±s)	Extracurricular sports (mean±s)	Sport club (mean±s)	Unorganized sports (mean±s)	Total amount of sports (mean±s)
		N = 3,660	N = 3,660	N = 3,617	N = 3,592	N = 3,550
Low SES	m	72.8±38.9	21.9±38.3	93.1±145.2	76.8±144.3	266.0±219.3
	f	69.3±39.6	17.9±38.7	46.7±74.6	34.0±98.6	168.8±144.6
	Ø	71.2±39.2	20.0±38.5	71.1±119.4	55.9±125.9	218.2±192.5
Mid SES	m	73.4±38.7	16.5±39.1	114.0±125.7	50.7±115.6	255.8±200.5
	f	73.7±33.9	15.4±39.5	83.9±112.1	55.6±141.3	228.2±1966
	Ø	73.6±36.4	16.0±39.3	98.9±120.0	53.1±128.9	242.1±199.0
High SES	m	72.3±32.1	18.1±43.7	144.3±150.9	49.9±101.7	285.4±204.7
	f	72.2±26.8	12.5±33.4	112.2±120.7	47.0±93.0	242.9±148.7
	Ø	72.3±29.8	15.6±39.4	129.7±138.8	48.6±97.8	265.9±182.3
ANOVA: sex		F = 0.5; p = .49;	F = 6.6; p = .01;	F = 34.3; p < .01;	F = 4.5; p = .03;	F = 43.7; p < .01;
		p.Eta^2^ = .000	p.Eta^2^ = .003	p.Eta^2^ = .015	p.Eta^2^ = .001	p.Eta^2^ = .013
ANOVA: SES		F = 1.6; p = .21;	F = 4.6; p = .01;	F = 50.6; p < .01;	F = 0.5; p = .60;	F = 8.5; p < .01;
		p.Eta^2^ = .001	p.Eta^2^ = .002	p.Eta^2^ = .020	p.Eta^2^ = .000	p.Eta^2^ = .005
ANOVA: sex*SES		F = 0.8; p = .45;	F = 2.4; p = .10;	F = 0.7; p = .48;	F = 10.6; p < .01;	F = 7.3; p < .01;
		p.Eta^2^ = .000	p.Eta^2^ = .001	p.Eta^2^ = .000	p.Eta^2^ = .006	p.Eta^2^ = .004

m: male; f: female, Ø: mean of males and females; s: standard deviation; SES: socioeconomic status

## 4 Discussion

The MoMo Wave 2 data confirm the trend of decreasing unorganized PA and increasing organized PA among children and adolescents living in Germany. The results are consistent with the results of Wave 1 (2009–2012) data [8]. However, a slight overall increase in PA that has been found in the data from 2003 to 2012 [8] could not be confirmed. Additionally, a closer look at the settings and disadvantaged groups revealed significant, setting-specific socioeconomic inequalities in PA behavior that should be used to tailor target-group and setting-specific interventions.

### 4.1 Organized physical activity

Whereas time spent in curricular sports remained stable from 2003 to 2017, the overall time spent in extracurricular sports increased from 6.1 to 16.6 minutes per week. This finding verifies the trend we observed from Wave 1 data [8], evolving extracurricular activities to an important setting for organized PA in Germany. One reason for this development might be the extensive implementation of daytime schools in Germany in the last years. The fear of a "PA cannibalism" at the expense of PA in sports clubs with increasing PA in schools and other extracurricular activities [26] is not confirmed. However, this study shows evidence for a general PA cannibalism, but at the expense of unstructured PA. Boys reported slightly higher amounts of extracurricular PA in school compared to girls, especially at the age of 11 to 13, but mean differences between genders are small. Taking into account the apparent gender inequality in sports clubs, extracurricular sports could be used as a vehicle to promote PA among girls in the school setting.

Considering the 15-year trend from Baseline to Wave 2, an increase in participation rates from 53.5% to 60.0% and 89.4 to 99.1 minutes PA in sports clubs was observed. Official numbers from the DOSB also report relatively stable rates of sports club memberships during the 2000s and 2010s with rates peaking at 79.8% among 7-to-14-year-old boys and 61.1 among 7-to-14-year-old girls [10]. In sum, a total of 60.0% of children and adolescents living in Germany report to participate in PA in sports clubs (Table 1). Overall rates of participation in sports clubs peak as early as between the age of six to ten and then decline slowly until adulthood. This decline in PA participation during adulthood is also reported in Canadian studies [27, 28]. Studies with non-humans show that a decline in PA of up to 50% appears at the maturation from adolescence to adulthood in nearly every living being [29]. However, Farooq and colleagues stated in a recent study that PA declines as early as from the age of 7 years in western civilizations and that there was no evidence indicating a substantially smaller decline during childhood than during the transition between adolescence and adulthood [30]. Considering the overall average time spent with PA per week (Table 2) compared to PA participation rates, a decline is delayed to late adolescents, as training volumes increase with age which, in turn, compensate for drop-outs. However, sports clubs are still the most important setting for the PA of children and adolescents in Germany and become more important worldwide [31]. A critical question is how to foster the active participation of adults in sports clubs. Offering a broader range of sports, including new trend sports and sports-related activities, as well as age-appropriated forms of motivation and competition are just some of many possible examples for interventions in the setting sports club.

### 4.2 Unorganized physical activity & playing outside

Data from Wave 1 showed that in Germany, unorganized sports activity as well as playing outside decreased between 2003 and 2012. This decline could be confirmed by our recent Wave 2 data. Other international studies also reported a declining trend in active play [32]. Reviews state that active, unstructured play in developed countries is decreasing for various reasons, including increased screen time, safety concerns (e.g. traffic, stranger danger), emphasis on organized youth sports, and parental work [5]. Although studies show that PA, particularly unorganized activity, is an effective way to decrease obesity in children [33], active play is likely of light intensity and to date, still, the significance of light and/or incidental PA among children and adolescents, is widely unknown [5]. One promising approach in promoting unorganized PA such as playing outside or active commuting is to improve walkability [34]. Walkability is, in turn, linked to unorganized sports activity and outside play in children [34]. Even though, there is some evidence that active commuting is not, or even negatively linked to organized PA in children under 10 years of age [35]. However, it may be a source of unorganized PA for older children and adolescents, just because of the fact they cross and use playgrounds on their way [36].

### 4.3 Overall PA & socioeconomic inequalities

Our study shows that overall PA among children and adolescents in Germany did not decline significantly during the 2000s and 2010s, although this expectation was expressed in previous research [37]. Other national studies and previous research support this finding [7, 8]. Referring to international PA recommendations [4] overall PA is on an unsatisfying level in Germany [38]. Nevertheless, the signaled crisis in childhood PA [5, 6] did not get worse during the 2000s and 2010s in Germany and there is a lack of representative data from periods before 2000 to discuss earlier trends.

However, as our survey assessed mainly sports activity, shifts in light PA may have been unobserved in our study. A recent Norwegian study among a representative sample of 9-year-olds found, that the prevalence of children and adolescents meeting the Norwegian PA recommendations was similar in 2005–2006 and 2011–2012. Yet, their accelerometer data indicated that both children and adolescents substituted time spent in light PA for time spent sedentary [39].

The decrease of unorganized PA among children and adolescents since the beginning of this study is alarming and needs to be observed meticulously. It is yet unclear, whether the lack of unorganized PA can be compensated in organized settings with the expertise of trained instructors. Although, participants in sports clubs typically spend 40–50% of their time in moderate to vigorous physical activity (MVPA) [40] and intensities of PA during organized sports are relatively high compared to other settings [14, 41]. Current studies indicate that sports clubs become a more and more important source of PA all over the world [31] and sports club participants are more likely to meet PA recommendations (OR 2.4–6.4, [31]). The question of the pros and cons of a mainly structured and guided PA at the expense of unorganized playing outside becomes increasingly important and should be the focus of further experimental research.

Although there is not much change in the *total amount* of PA, previous research that focused on biological and environmental correlates [42], different settings [8], and socio-structural factors [43] revealed that there is social and environmental inequality in PA attendance and PA behavior in Germany. The SES has been identified as an important correlate of PA and a healthy lifestyle [5, 34]. Socioeconomic disparities in health behavior have been found by numerous studies all over the world and account for a large amount of PA inequality [44–46]. By analyzing different PA-relevant settings, the present study shows that these socioeconomic differences are not evenly pronounced in each setting. For example, there is no gender gap in curricular sports, and gender differences in extracurricular sports are small and age-dependent (Table 2). Adding the SES as a factor, our study shows that a prevalent gender gap in unorganized PA is mainly due to differences between gender among youths from families with low SES (Table 3) and can therefore not be generalized. In families with an intermediate SES, girls do even more unorganized PA compared to their male counterparts. The fact that different social groups participate in different types of settings provides the opportunity for target-group-specific interventions. An example would be the development of additional programs for extracurricular sports in schools utilizing cooperations between schools and sports clubs to reach out especially for girls from low SES families or girls with migration backgrounds. On the other hand, these setting-specific trends sharpen the scope of shifts in the importance of those settings. For example, the shift from unorganized PA to PA in sports clubs may leave boys from low SES families with lesser overall PA.

The large meta-analysis from Althoff et al. (2017) about PA data from smartphones found these inequalities in PA to be crucial for the prevalence of inactivity driven diseases such as overweight and obesity.

### 4.4 Strength and limitations

The present study is limited to its observational nature and we do not intend to infer causality from paralleled trends or significant correlations. The main goal of MoMo is to track and report PA and fitness of children and adolescents in a nationwide sample, and significant effort was put into collecting representative data from 167 sample points all over the country.

PA was assessed by self-reports. This method has various limitations including recall bias and social desirability. Measuring PA by objective methods such as accelerometers is more accurate in most types of PA, but is always limited to a short time interval, and unless a diary is added, the setting in which the PA took place is not captured. Since accelerometers capture any form of PA in a specific time frame, the correlation with self-reported habitual PA in specific settings is expected to be restricted, even when summarized. An accelerometer is also a very responsive tool towards socially desired behavior, and children drop it for some sports like swimming, martial arts, and sometimes even curricular sports in school. Using a questionnaire offered the chance to assess different types of exercise as well as other PA parameters like setting and sports club membership during a normal week, even when the person is, for example, on vacation or ill.

## 5 Conclusions & forecast

The MoMo Wave 2 data show an ongoing shift from unorganized PA (unorganized sports & playing outside) to organized PA (school & sports club) among children and adolescents in Germany. Contrary to many expectations, the introduction of smartphones and social media platforms during the 2000s did not affect the overall PA of youth in Germany as measured by questionnaire. MoMo is an observational study and we cannot conclude the underlying cause for reported trends, but the declining amount of time spent in unorganized PA should be further observed. To picture the overall amount of PA including PA with light intensity in the future, we included the use of accelerometers in Wave 2 and the ongoing Wave 3 and will add those data to our analyses in the future.
